# A Questionnaire Elicitation of Surgeons’ Belief about Learning within a Surgical Trial

**DOI:** 10.1371/journal.pone.0049178

**Published:** 2012-11-08

**Authors:** Jonathan A. Cook, Craig R. Ramsay, Andrew J. Carr, Jonathan L. Rees, UKUFF trial group

**Affiliations:** 1 Health Services Research Unit, University of Aberdeen, Aberdeen, United Kingdom; 2 Nuffield Department of Orthopaedics, Rheumatology and Musculoskeletal Science, and the National Institute for Health Research Biomedical Research Unit, University of Oxford, Oxford, United Kingdom; Cardiff University, United Kingdom

## Abstract

**Introduction:**

Surgeons gain expertise as they repeatedly conduct a procedure. Such learning is widely acknowledged to pose a challenge to evaluating new surgical procedures. Most surgical trials report little if any information on learning. We elicited surgeons’ belief regarding learning within the context of a randomised trial which assessed two surgical procedures.

**Materials and Methods:**

Surgeons participating in the UKUFF trial were sent a postal questionnaire requesting details on current practice, prior experience and their belief regarding acquiring proficiency and the learning curve of operation time for two surgical procedures (open and arthroscopic rotator cuff repair).

**Results:**

In total 52 (58%) participating surgeons returned a completed questionnaire. The median (IQR) number of procedures required to acquire proficiency were 17 (10,23) and 35 (23,50) for the open and arthroscopic repairs respectively. The distribution of surgeons’ belief regarding the initial point had median (IQR) of 109 (69,128) and 145 (97,171) minutes for open and arthroscopic repair respectively. Corresponding values for the plateau point were 60 (46, 82) and 79 (58, 110).

**Conclusions:**

We have shown that information on the current practice, prior experience and beliefs on the learning process of a surgical procedure can be elicited using a short questionnaire. The approach could aid the interpretation of trial results in terms of generalisability and be used a priori in the design of a trial.

## Introduction

Surgeons are widely acknowledged to gain expertise as they repeatedly conduct a procedure. This change in performance over time (a learning curve) can be an impediment to conducting and interpreting surgical randomised controlled trials (RCT) [1]. A RCT of a new procedure can be delayed (perhaps indefinitely) as surgeons may still be learning the procedure and view any evaluation as ‘unfair’ - inexperienced versus experienced surgery. Even upon completion of a surgical RCT, the results may be criticised as biased if the levels of expertise were not explicitly measured.

Two general approaches to addressing the impact of learning in an RCT have been proposed: a design and an analysis strategy. Under the design strategy, the eligibility of participating surgeons is considered against a threshold of expertise (e.g. a surgeon must have performed at least 10 cases and supervised in a further 5 cases) [2]. In a trial where procedures are conducted only by those with “expertise” in that procedure (e.g. an expertise-based trial), sufficient expertise must be defined [3]. Limited if any empirical data may be available to justifying a particular specification of “expertise”. In practice, this may be left to a surgeon’s own judgement. Systematically reviewing the literature has been proposed to quantify the effects of learning though this approach is limited by the poor general level of reporting of expertise information [4]. Alternatively, under an analysis strategy, a RCT may be conducted with the expectation that assessment of the impact of learning on trial results will be undertaken in the statistical analysis at the end of the study [1], [5], [6]. Such an approach is likely to have high data requirements and may only be a realistic option for large RCTs. A formal approach to eliciting expertise may provide an alternative solution. Methods for eliciting beliefs in general were recently systematically reviewed though it was not viewed possible to recommended a particular method [7]. To our knowledge, no formal Bayesian elicitation of surgeon belief about expertise and learning has been conducted. This study aimed to elicit surgeons’ belief regarding learning within the context of a randomised trial which assessed two surgical procedures. The specific objectives were:

1. Elicit surgeons’ belief on the number of cases a surgical trainee requires to gain proficiency in open and arthroscopic rotator cuff repair and
2. Elicit surgeons’ belief on the shape of the respective learning curves for operation time.

**Figure 1:**
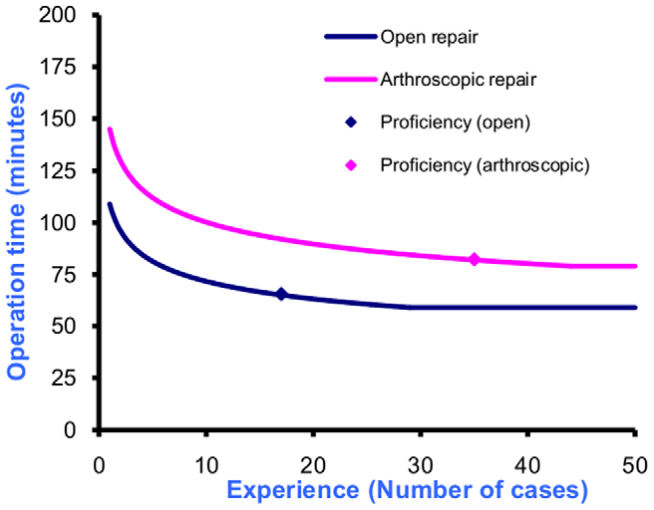
Learning curve of operation time for open and arthroscopic rotator cuff repair (mean aggregation).

**Table 1:** Learning curve (parameters) of operation time for open and arthroscopic rotator cuff repair.

Aggregation method	Parameter	Open repair Valid N Median (IQR)		Arthroscopic repair Valid N Median (IQR)	
**Mean**	**Initial point (min)**	32	109 (69,128)	29	145 (97,171)
	**Plateau (min)**	38	60 (46,82)	35	79 (58,110)
	**Plateau (number of cases)**	29	29	24	44
**Geometric mean**	**Initial point (min)**	32	105 (65,123)	29	143 (94,169)
	**Plateau (min)**	38	55 (43,78)	35	77 (56,107)
	**Plateau (number of cases)**	29	24	24	39

## Methods

The UKUFF trial was a multicentre RCT comparing three interventions: rest and exercise management, open surgical repair and arthroscopic surgical repair for participants with a tear of the rotator cuff (ISRCTN97804283) and a target recruitment of 690 participants. Participating surgeons specified whether they were willing to conduct open repair only, arthroscopic repair only, or both surgical procedures. Participation in the UKUFF trial was restricted to consultant orthopaedic surgeons in the UK with a minimum of two years experience in consultant practice and who performed a minimum of 5 rotator cuff repairs per annum. This reflected a pragmatic view of surgeons who currently undertake the procedure in routine clinical practice. Surgeons were sent a postal questionnaire (see appendix S1) from the UKUFF trial office as they were recruited. Ethical approval was granted by Oxfordshire research ethics committee C (REC reference number: 07/Q1606/49) in the UK. The short questionnaire was accompanied by a letter with the trial letterhead requesting details regarding their current practice, prior experience, their belief regarding a surgical trainee acquiring proficiency and the learning curve of operation time for both surgical procedures.

The elicitation process followed a variable interval method with median and interquartile range (IQR) points requested for the distribution of the number of cases required to acquire proficiency for both procedures [7]. Under the variable interval method, a finite number of points are taken to fix the underlying subjective distribution of belief. A composite question/graphical method was used to elicit the shape of the learning curve for operation time. For both open and arthroscopic procedures, two learning curve parameters (first procedure and plateau level) were elicited in written form and the shape of learning curves displayed graphically. Two reviewers independently categorised the shape of the curve and elicited values for the initial point and the plateau point of the learning curve. The surgeons’ beliefs about learning were combined to produce a summary distribution using a mathematical average approach (mean aggregation). A sensitivity analysis on this approach combined responses using a geometric mean of individual responses [8]. Under both approaches individual responses received equal weight. Only values from surgeons who provided a) values for all three distribution points (median and IQR limits) and b) coherent estimates were used to form the summary distributions. A learning curve was generated using the most common shape and a power law curve fitted which has theoretical justification as representing learning[9]–[11]. Pre-specified analyses contrasted surgeons’ belief about the two procedures using a paired sign test using 5% level (two sided) as a marker of statistical significance.

## Results

In total, 52 (58%) participating surgeons returned a completed questionnaire representing 42 (68%) of centres. Of those returned, 21 surgeons performed only open surgical repair, 11 only arthroscopic repair and 20 both open and arthroscopic repairs. The median (IQR) number of cases previously performed across all surgeons were 100 (40,200) and 45 (7,100) for the open and arthroscopic repairs respectively. Corresponding values for the number of cases typically performed in a year were 9 (3,24) and 8 (0,23) respectively.

The summary distribution of surgeons’ belief regarding the number of cases required (for a trainee) to acquire proficiency had median (IQR) of 17 (10,23) and 35 (23,50) cases respectively for the open and arthroscopic procedures. Only 3 (6%) and 2 (5%) responses respectively were not coherent. Corresponding values for the sensitivity analysis were similar with median (IQR) of 14 (8,19) and 30 (18,41) cases respectively for the open and arthroscopic procedures. Individual surgeon responses for the median point ranged from 5 to 50 for open and 10 to 100 cases for arthroscopic procedures. Proficiency estimates for arthroscopic procedure was significantly different at 5% significance level (paired sign test) when compared within surgeon (N = 38; p<0.001 for all three distribution parameters). Surgeons who carried out the arthroscopic procedure suggested less cases were required to acquire proficiency for both the open [14 (7,19) versus 22 (14,28) - median (IQR)] and arthroscopic [32 (19,46) versus 42 (32,59) - median (IQR)] procedures compared to surgeons who did not. Overall, the distributions suggest substantial variation amongst trainees in acquiring proficiency for both procedures.

The shape of the learning curve of operation time was provided in 92 graphs (49 and 43 respectively for open and arthroscopic procedures). The shape of the graph was categorised as a concave decay curve for 32 (35%), 29 (31%) as S-shaped decay curve, 22 (24%) as straight line decay and 9 (9%) comprising of others shapes. Proposed shapes were generally similar for the open and arthroscopic procedures. The summary distribution of surgeons’ belief (mean aggregation) regarding the initial point had median (IQR) of 109 (69,128) and 145 (97,171) minutes for open and arthroscopic repair respectively (Table 1). Corresponding individual responses for the median initial point ranged from 40 to 175 minutes for open and 102 to 200 minutes for arthroscopic procedures. Values for the sensitivity analyses were similar. Corresponding values for the plateau were 60 (46, 82) and 79 (58, 110) minutes for which individual responses for the median plateau time ranged from 15 to 130 and 48 to 115 minutes. However, the proportion of non-coherent values (i.e. median operation time not within IQR) was substantial between 7 (17%) and 15 (32%). As with the proficiency estimates, surgeons estimated the arthroscopic procedure to have a higher operation time than the open procedure for both the initial point (N = 24; p<0.001 for all three distribution parameters) and plateau point (N = 28; p≤0.001 for all three distribution parameters). The elicited shapes and proficiency points (using the median values) are graphically displayed in Figure 1.

## Discussion

We have shown that information on the current practice, prior experience and beliefs on the learning process of a surgical procedure can be elicited using a short questionnaire. The approach could aid the reporting and interpretation of a surgical trial, specifically the generalisability of its results. Concerns regarding the attribution of a trial’s results to routine surgical practice, where one of the procedures is skill dependent, is common. Reporting on the prior expertise of the surgeons participating in a trial, and the beliefs regarding the impact of learning, could aid the process of assessing to whom the trial is most applicable and the likelihood of expertise impacting upon the trial result. Alternatively, the questionnaire could be sent to surgical participants *a priori* to allow the information to be used to aid the design of a trial (eg setting the requirements for surgeon participation in the trial) [12]. A possible extension is the formal use of this information in the trial statistical and/or economic analyses. We used two different elicitation approaches to capture belief relating to the learning curve – question and composite graphical/question approach. The approaches elicited the number of case to acquire proficiency and the initial point, plateau point and shape of the learning curve. A distribution, as opposed to a single estimate, of surgical trainee learning was elicited; acknowledged that surgical trainees will likely learn at different rates [13]. The elicited learning curves could potentially be used to assess the robustness of the trial results to differing learning assumptions and could be used in an economic evaluation.

The study had several strengths – the sample size was relatively large for elicitation studies, the approach was grounded in a theoretical approach (Bayesian), the results were consistent with other approaches but added further information on the differences in learning between trainees, and finally the method is relatively straight forward to use. In the example, learning was measured using the proxy of the number of cases performed in a particular intervention. While this is known to have its limitations [1], a more precise measure of learning has yet to be determined. Therefore, while empirical data on proficiency and learning is preferable for trial design, it is often sparse or inconclusive [12]. For surgical trials, and other trials evaluating operator-dependent interventions, this approach could provide a more robust basis for such a choice.

There are a number of limitations to this study. As we elicited beliefs about learning, personal experience and preferences, and attitudes will have influenced the responses. Additionally, whereas the proficiency approach had a high response rate and internal validity, the combined graphical/question approach suffered from incomplete and inconsistent responses in some cases (eg the initial point from the graph was not within the IQR). Clearer framing of the method, defining concepts (eg proficiency), the use of feedback and/or more extensive questionnaire could improve inconsistent responses but may reduce the response rate. Nevertheless, the response rate is in-line with other postal studies for health professionals [14]. Furthermore, the results were consistent with other studies on learning arthroscopic shoulder repair which suggest that proficiency could be gained within 50 cases [15], [16]. This consistency provides some reassurance regarding external validity. Comparison of the surgeons’ belief to outcome data collected as part of the UKUFF trial would also allow assessment of this. We elicited the learning curve for operation time, which though intuitive for elicitation, is typically of limited clinical important.

Learning curves continue to be viewed as an impediment to RCTs of operator dependant interventions such as surgical procedures. Study design and analyses accounting for learning curves are often suboptimal and arbitrary. The questionnaire approach used here allowed estimates of proficiency and learning curves with associated distributions along with surgeons’ expertise. Such an approach could be viewed as attractive when empirical data is sparse if it has good internal and external validity. The learning curve is likely to vary between surgical procedures and across surgical specialties [13], [17] and further evaluation is needed before the merit of this approach can be concluded.

## Supporting Information

Appendix S1 **Surgeon questionnaire.**
(DOC)
