# Supplementary material for: A Questionnaire Elicitation of Surgeons’ Belief about Learning within a Surgical Trial
Source: PLoS One. 2012 Nov 8;7(11):e49178. doi: 10.1371/journal.pone.0049178 (PMC3493499; doi:10.1371/journal.pone.0049178)
Supplement: Appendix S1 — Surgeon questionnaire. (DOC) [file pone.0049178.s001.doc]

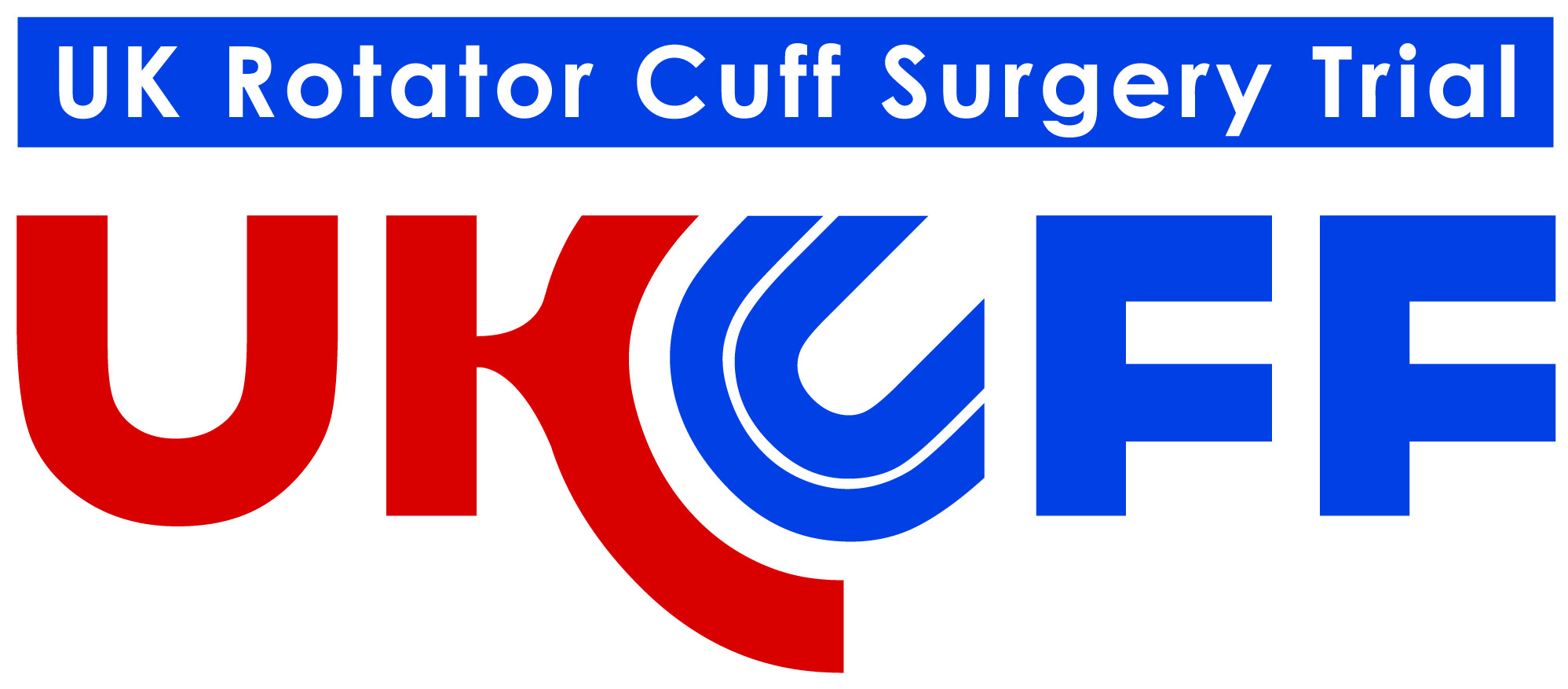
**Surgeon questionnaire**

**LEARNING CURVE FOR ROTATOR CUFF REPAIR**

Carrying out clinical trials in the field of surgery is very difficult and the presence of a learning curve may confound trial results making our speciality less appealing to funding bodies and journals. The aim of this questionnaire is for you to express ***your*** belief about the learning curve related to surgery for repairing tears of the rotator cuff. We are interested in ***both*** open (including mini-open) and arthroscopic surgical repairs. This questionnaire will help with the design of future surgical trials and has been sent to you with the full approval of the UKUFF Trial group.

**GENERAL INFORMATION**

**Quest No.**

┕┷┷┙

**1.** What is your clinical position? __________________________

**2.** Do you carry out both open and arthroscopic repair of the rotator cuff?

Both/Open repair only/Arthroscopic repair only *(please circle your choice)*

**3.** How many cases of rotator cuff repair surgery would you typically perform in a year?

**a)** **Open repair**  ________ cases

**b)** **Arthroscopic repair** ________ cases

**4.** How many cases of rotator cuff repair surgery have you performed in your career?

**a)** **Open repair**  ________ cases

**b)** **Arthroscopic repair** ________ cases

**SURGICAL TRAINING**

For a typical orthopaedic trainee who has completed higher surgical training in orthopaedics and has observed rotator cuff repair**:**

**5.** How many procedures’ experience do you think is required to acquire proficiency in this surgical operation?

**a)** **Open repair**  ________ cases

**b)** **Arthroscopic repair** ________ cases

**What is an interquartile range?**

The interquartile range varies from the number you would expect 25% of surgical trainees to require to the number that would cover 75% of surgical trainees. For example, an interquartile range of 5 to 10 procedures’ experience would imply you believed that 25% of surgical trainees needed 5 or less procedures’ experience and 75% of trainees would require 10 or less procedures’ experience. Similarly, an interval of 35 to 150 procedures’ experience would state your belief that 75% of trainees need no more than 150 procedures’ experience whereas 25% of trainees required 35 or less.

**6.** What is your estimate of the corresponding *interquartile range*? *(see box above)*

**a)** **Open repair** *interquartile range* ________ to ________ cases

**b) Arthroscopic repair** *interquartile range* ________ to ________ cases

**Please Turn Over**

**OPERATION TIME**

**7.** Please draft a curve on the plot below to reflect your belief in how long the operation will take as a surgical trainee gains experience in open and arthroscopic repair. For example, a horizontal line would state that you do not believe the operation time will change as experience is gained. Any downward curve would state the operation time *reduced* as experience *increased.* **Please fill-in the values in the x-axis to match your curve.**

**a) Open Repair b) Arthroscopic Repair**

**8.** For the first procedure performed, please give an estimate of the corresponding *interquartile* range for the operation time?

**a) Open** **repair** *interquartile range* ________ to ________ minutes

**b) Arthroscopic** **repair** *interquartile range* ________ to ________ minutes

**9.** For a procedure performed once a surgeon’s performance has plateaued, please give an estimate of the corresponding *interquartile* range for the operation time?

**a) Open** **repair** *interquartile range* ________ to ________ minutes

**b) Arthroscopic** **repair** *interquartile range* ________ to ________ minutes

**COMMENTS RELATED TO LEARNING CURVE**

**10.** Please use the space below to provide any additional information you feel is relevant to learning surgical repair for rotator cuff tears
